# Application of chitosan-ZnO nanoparticle edible coating to wild-simulated Korean ginseng root

**DOI:** 10.1007/s10068-022-01054-7

**Published:** 2022-03-29

**Authors:** Soo Hyun Kang, Hee Jin Cha, Seung Won Jung, Seung Ju Lee

**Affiliations:** Department of Food Science and Biotechnology, Dongguk University-Seoul, Ilsandong-gu, Goyang-si, Gyeonggi-do 10326 Republic of Korea

**Keywords:** Wild-simulated Korean ginseng root, Edible coating, Zinc oxide, Chitosan, Shelf life

## Abstract

Chitosan-ZnO nanoparticle (ZnONP) edible coating was applied to extend shelf life of wild-simulated Korean ginseng root (WsKG). In antimicrobial testing of various coating solutions (0.01, 0.02, 0.03% ZnONP), *Bacillus cereus* (Gram-positive) and *Escherichia coli* (Gram-negative) were most inhibited by the 0.03% chitosan-ZnONP solution. The 0.03% chitosan-ZnONP solution was finally used for edible coating of WsKG. In SEM analysis, the coat of chitosan and ZnONP was well-formed on the surface of WsKG. In isothermal storage tests (temperature: 5–20 °C, RH: 95%), microbial limit (4.70 log CFU/g) of total aerobic bacteria for non-coated and coated WsKG were reached at 3.9 and 6.3 weeks at 5 °C, 1.9 and 4.3 weeks at 10 °C, and 1.3 and 2.0 weeks at 20 °C, respectively. Mold occurred in the non-coated sample at 4 weeks at 5 °C, but not in the coated sample during 6 weeks. Chitosan-ZnONP edible coating was very effective in preserving WsKG.

## Introduction

Wild-simulated Korean ginseng root (*Panax ginseng* C.A. Meyer; WsKG) is a botanical plant belonging to the genus *Panax* within the family Araliaceae (Kim et al., 2019). WsKG is a forest product with various human health benefits, such as lowering blood pressure, anti-cancer, antioxidant, liver detoxification, and lowering lipids (Hong et al., 2008; Kim and Kim, 2006; Kim et al., 2004; Kwon et al., 2003; Yun et al., 2004). The plant presents a higher ginsenoside content compared with ginseng and wild ginseng (Blix et al., 1957), and it was recognized as the second most effective in terms of pharmacological activity after natural wild ginseng (Kim et al., 2007). However, it is one of the forest products that are prone to deterioration due to its high moisture content (Chun and Song, 2007; Kim et al., 2002). Therefore, it is important to minimize the quality loss of WsKG during the distribution process until it is delivered to the consumer.

Ginseng is generally packaged in a cardboard box or polypropylene bag for wholesale, and a polyethylene bag or a box made of manila cardboard at the consumer stage (Park et al., 2000). However, these methods are insufficient to maintain product quality. Deterioration can be suppressed when controlled atmosphere and modified atmosphere packaging methods are used (Hu et al., 2004a; Jeon and Lee, 1999; Kwon et al., 1994). Lee et al. (1976) observed changes in skin and tissue quality of vacuum-packed ginseng roots when fresh ginseng was frozen at −40 °C and stored at −20 °C for 3 months. Jin et al. (2017) extended the shelf life of fresh ginseng roots to 38 weeks by combining sanitizer washing, edible coating (0.5% chitosan and three organic acids), and modified atmosphere packaging.

An antimicrobial coating reduces the exchange of water, O_2,_ and CO_2_ between food and the environment, and inhibits respiration and oxidation reactions (Min and Krochta, 2005; Robles-Sánchez et al., 2013). This food preservation methods improve the stability, quality, and functionality of food by interfering with the growth of unfavorable microorganisms in the food during storage, transportation, processing (Mellinas et al., 2016). Application of chitosan coatings to fresh food has proven successful in delaying the ripening of strawberries (Hernández-Muñoz et al., 2006), as well as slowing the respiration and browning extent of mushrooms (Liu et al., 2019). Chitosan is well recognized for its intrinsic antimicrobial activity against fungi and some bacteria (Doores, 1993; 2002; Kong et al., 2010; Romanazzi et al., 2013). However, the single chitosan-based coating has the disadvantage of inhibiting only limited microorganisms (Ravi, 2000). Other limitations of chitosan are its low mechanical, barrier and processing properties, and high cost compared with plastic films (Tunc and Duman, 2011).

Various metal nanoparticles (NP), such as silver, gold, titanium oxide, copper oxide, magnesium oxide, and zinc oxide (ZnO), exhibit antibacterial properties (Beyth et al., 2015; Zhang, 2015). ZnONP have been used in the food packaging industry because of their antibacterial properties against a wide range of microorganisms (Espitia et al., 2013; Li et al., 2009). ZnONP can be mixed with a polymer film to prepare antibacterial nanocomposite packaging films that preserve food by preventing the growth of microorganisms on the food surface (Espitia et al., 2013; Soares et al., 2009; Yu et al., 2009). Highly reactive oxygen species, such as peroxide ions, hydrogen peroxide, singlet oxygen, and hydroxyl and superoxide radicals are generated in the presence of ZnONP (Li et al., 2009; Paisoonsin et al., 2013; Tankhiwale and Bajpai, 2012). The production of hydroperoxide can disrupt microbial homeostasis and destroy it. ZnO is partially dissociated by acetic acid to form zinc ions (Zn^2+^). This partially dissociated Zn^2+^ also plays a role in antibacterial activity by disturbing the homeostasis of microorganisms and entering the cells, causing cytotoxicity (Pasquet et al., 2014). ZnO nanocomposite packaging has been effectively applied to kiwifruit, apple, mango, and papaya to extend the shelf life (Lavinia et al., 2020; Li et al., 2011; Meindrawan et al., 2018; Meng et al., 2014). Besides, acting as an additive in food, ZnO has been applied to supplement and strengthen nutrients (Shi and Gunasekaran, 2008; Suyatma et al., 2014).

In this study, chitosan-ZnONP edible coating was newly applied to extend shelf life of the WsKG. First, an antimicrobial experiment was conducted to determine the ZnO concentration of the coating solution. The aggregation of ZnONP undesirable for the antimicrobial effect, was examined through particle size analysis. The distribution of chitosan and ZnONP in the WsKG coating was analyzed by SEM. Finally, the antimicrobial effect of the chitosan-ZnONP coating was evaluated based on general bacteria analysis and observation of appearance of WsKG during storage.

## Materials and methods

### Materials

The WsKG used in this study was 6-year-old root grown in Sansam Village (Wanju, Korea). Chitosan (75–85% deacetylated), acetic acid solution, and ZnONP (average particle size of 20 ± 5 nm) were purchased from Sigma-Aldrich (Seoul, Korea). K_2_SO_4_ and CaCl_2_ were purchased from Daejung chemicals & Metals Co. (Siheung, Korea). *Escherichia coli* ATCC 25,922 and *Bacillus cereus* ATCC 11,778 were brought from the American Type Culture Collection (ATCC, Seoul, Korea). *E. coli* and *B. cereus* were cultivated at 37 °C for 16 h by picking one inoculation loop and spreading on Luria–Bertani (LB) medium (BD DIFCO, New Jersey, USA). Microbial stock was prepared by inoculating LB broth and 50% glycerol (6:4 v/v) with the bacteria, which was stored at −80 °C.

### Chitosan-ZnONP coating of WsKG

The chitosan-ZnONP solution was prepared by referring to Kanmani and Rhim (2014) with modifications. Briefly, 0.03 g ZnONP was dissolved in 0.1 L of 1% acetic acid solution. In another flask, 3 g chitosan was dissolved in 0.3 L of 1% acetic acid solution at 60 °C. The two solutions were mixed and stirred at 80 °C for 5 min, then cooled to room temperature and used for coating. WsKG washed with distilled water was immersed in a 1% CaCl_2_ solution for 5 min. Drained WsKG was immersed in the chitosan-ZnONP solution for 5 min, drained and left at room temperature for 4 h.

### Antimicrobial and physical characterization of chitosan-ZnONP solution and coating

The chitosan-ZnONP solution (0.01, 0.02, 0.03% w/v ZnONP) was added at 0.1% to LB broth and vortexed. A 50-mL aliquot of the LB broth was added to a 100-mL Erlenmeyer flask, followed by 50 μL of the microbial stock, and the mixture cultured at 37 °C for 24 h with agitation at 200 rpm (× 1.4 g) in a thermoshaker incubator (Allsheng Instruments Co., Hangzhou, China). Microbial concentration was monitored by recording the optical density (OD_600_ nm) at certain intervals using a spectrophotometer (UV-1800, Shimadzu, Kyoto, Japan) (Porel et al., 2011). Results were reported as the average of three repeated measurement.

The particle size distribution and polydispersity index (PDI) of ZnONP were obtained using a zeta-potential analyzer (ELS-8000, OTSUKA, Tokyo, Japan) at 25 °C. Prepared by putting 1 mL of chitosan-ZnONP solution (0.03%) in an Eppendorf tube. Its measurement principle is based on dynamic light scattering (DLS) (Bhattacharjee, 2016). The incident beam was a helium–neon (He–Ne) ion laser at 630 nm. The refractive index and viscosity of the solution were 2.1 and 0.89 cP, respectively.

The coated and non-coated WsKG were observed under a SEM (Hitachi S-3000 N, Hitachi Instruments, Tokyo, Japan) operating at 20 kV and 180 × magnification to observe the microstructure of the chitosan-ZnONP coating. The coated and non-coated WsKG were placed in each Petri dish and evaporated at room temperature.

### Storage test of chitosan-ZnONP coated WsKG

Twenty WsKG (coated or non-coated) was stored over a K_2_SO_4_ saturated salt solution (95% RH, equivalent to humid storage condition) in a desiccator (internal diameter, 240 mm) at 5, 10, and 20 °C for 6 weeks. One WsKG from each storage condition was removed at certain intervals and placed in a sterile bag with peptone water (0.1% sterile peptone, w/v) of 10 times weight of the ginseng, and homogenized with a stomacher (Bagmixer®400, Interscience, Paris, France) for 90 s. The stepwise-diluted sample was dispensed into 3 M Petrifilm™ (aerobic count plate; 3 M, Seoul, Korea) and cultured at 37 °C for 48 h, followed by the enumeration of colonies (*n* = 3). The microbial concentration was expressed in log CFU/g units calculated by considering the dilution factor, the amount of peptone, and the weight of WsKG.

The appearance of WsKG was qualitatively observed with the naked eye, given in photographs.

## Results and discussion

### ZnONP size in coating solution

**Fig. 1:**
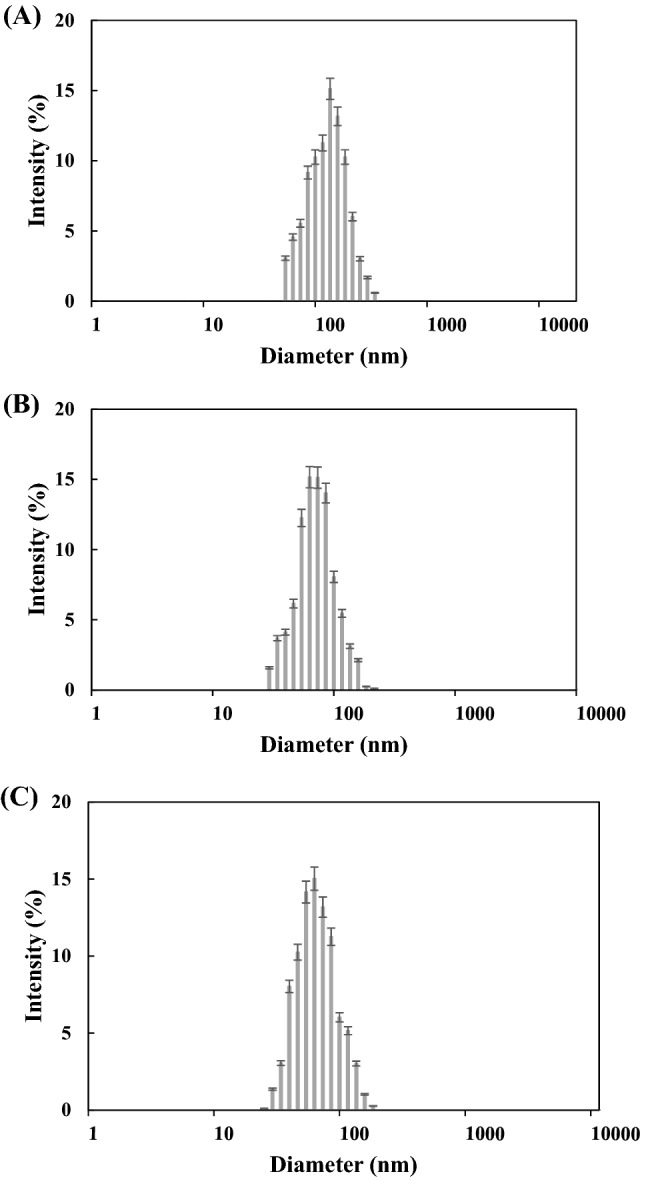
Distribution of ZnONP on the chitosan-ZnONP film according to particle size analysis. (**A**) 0.01% ZnONP; (**B**) 0.02% ZnONP; (**C**) 0.03% ZnONP

When ZnONP was dispersed at 0.01, 0.02, and 0.03% (w/v) in the acetic acid solution used for dissolving chitosan, the average particle size was 106.5, 94.2, and 92.7 nm, showing similar sized NPs (Fig. 1). It has been reported that the average particle size decreases as the concentration of ZnONP increases (Kołodziejczak-Radzimska et al., 2012). The original size before dispersion was 20 ± 5 nm but increased after dispersion in the solution because of the ZnONP aggregation (Shi and Gunasekaren, 2008). The ZnONP aggregation were also observed in the preparation of the films of ZnONP incorporated chitosan (Lavinia et al., 2020) and pectin (Suyatma et al., 2014). The ZnONP of 0.03% was considered to have the best antimicrobial effect because it was the smallest in particle size and therefore the largest in surface area favorable for its reactivity. According to Webster and Seil (2012), the specific surface area of nanoparticles increases with decreasing particle size, allowing greater material interaction with the surrounding environment. Therefore, for intrinsic antibacterial materials such as zinc and silver, increasing the surface-to-volume ratio enhances the antibacterial effect.

The PDI of ZnONPs of 0.01, 0.02, and 0.03% were 0.385, 0.251, and 0.116, respectively. It has been reported that the PDI decreases as the concentration of ZnONP increases (Lavinia et al., 2020). PDI means the degree of non-uniformity of a particle size distribution. PDI of < 0.1, 0.1–0.4, and > 0.4 indicate highly monodisperse, moderately polydisperse, and highly polydisperse, respectively (Bhattacharjee, 2016). Therefore, the ZnONPs were considered to have a moderate size distribution.

### Antimicrobial property of chitosan-ZnONP coating solution

**Fig. 2:**
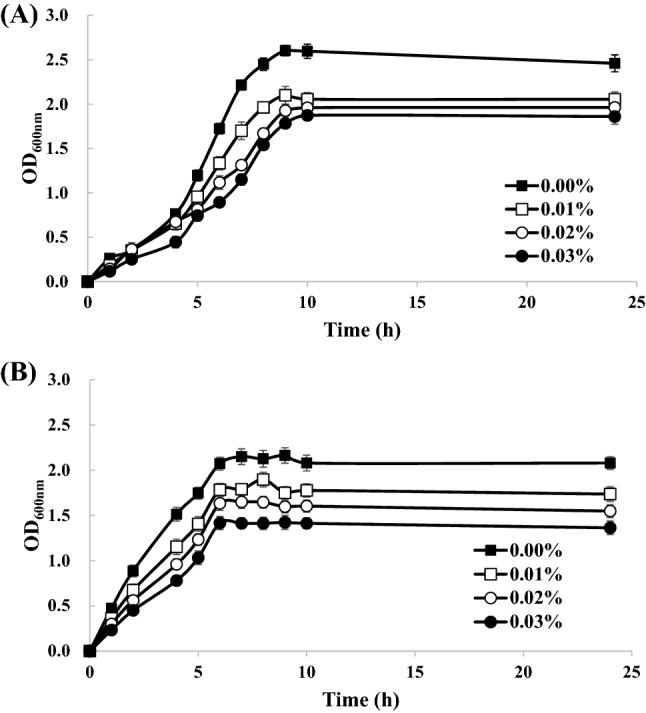
Microbial inhibition in LB broth inoculated with (**A**) *Bacillus cereus*, (**B**) *Escherichia coli* by different concentrations of ZnONP. Error bars represent the standard deviation (*n* = 3)

The microorganisms that can deteriorate ginseng are known as *B. cereus*, *E. coli*, *Staphylococcus aureus*, etc. (Kim and Yang, 2018). Among them, *B. cereus* and *E. coli* were applied to this study. As the amount of ZnO in the coating solution increased from 0.00 to 0.01, 0.02, and 0.03% (w/v), the maximum OD_600 nm_ (equivalent to microbial content) of *B. cereus* growth decreased from 2.60 to 2.04, 1.96, and 1.87, respectively (Fig. 2). In the case of *E. coli*, the maximum OD_600 nm_ were 2.16, 1.90, 1.65, and 1.42, respectively. As a result, it was found out that as the amount of ZnO increased, the growth of microorganisms was better inhibited.

*Bacillus cereus* and *E. coli* reached the maximum OD_600 nm_ after 9 and 7 h, respectively. *B. cereus* and *E. coli* are Gram-positive bacteria and Gram-negative bacteria. In Gram-positive bacteria, the cell wall is composed of multilayers of peptidoglycan with an abundance of pores through which the ZnO particles could enter the cells. By contrast, Gram-negative bacteria have a thin peptidoglycan layer, but a complex cell wall structure, which would be less susceptible to attack by NP (Anitha et al., 2012). Therefore, the NP have more efficacious antimicrobial activity against Gram-positive bacteria with simple cell walls than Gram-negative bacteria with complex cell walls (Anitha et al., 2012; Paisoonsin et al., 2013). In contrast, Rhim and Wang (2013) and Li et al. (2009) reported that the thick layer of peptidoglycan inhibited ZnONP from penetrating cells of Gram-positive bacteria, while ZnONP could easily penetrate the thin peptidoglycan layer of Gram-negative bacteria. In this study, the findings were consistent with the ZnONP being more effective against *B. cereus*.

### Microstructure of chitosan-ZnONP coating film

**Fig. 3:**
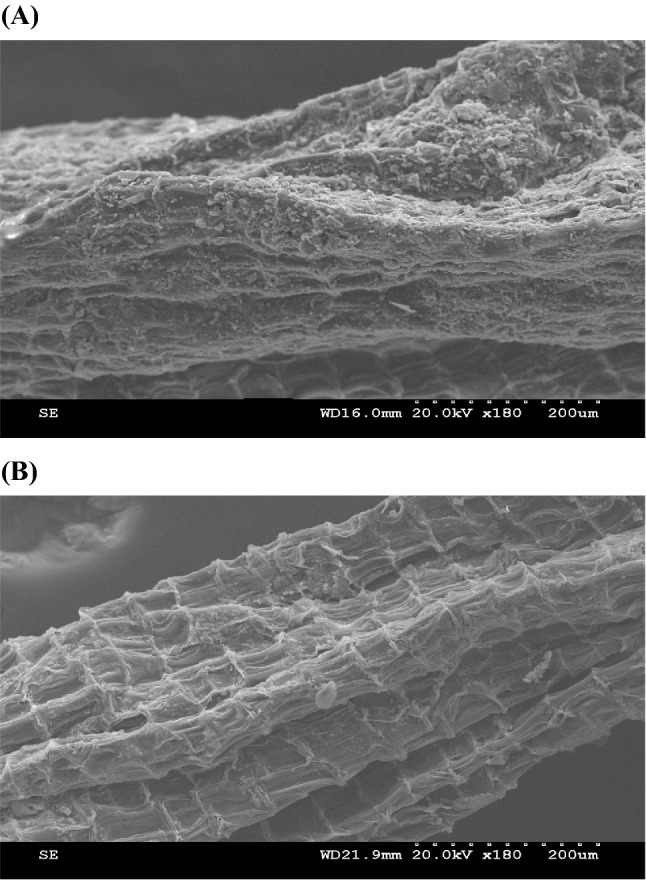
SEM images of (**A**) chitosan-ZnONP coated WsKG; (**B**) non-coated WsKG

SEM is used to study the surface morphology of the NP composite films (Akhtar et al., 2018). In the non-coated WsKG, a wrinkled surface corresponding to WsKG peel was observed, but the coated WsKG had agglomerated particles on its coat (Fig. 3). The coating thickness or content could not be presented, because, unlike foods such as apples, WsKG did not have a constant surface curvature and roughness, and its surface area was not constant even with the same weight, resulting in too much variation. In general, when metal NPs are identified through SEM, they are observed to be irregular and lumpy (Plascencia-Villa et al., 2012; Tailor et al., 2019). In contrast, chitosan appears to have a uniform shape without lumps (Paul et al., 2018). For instance, TiO_2_NP appears as agglomerated particles distributed on the chitosan matrix in the microstructure of chitosan-TiO_2_NP composite film (Xing et al., 2020). Accordingly, it was judged that the agglomerated particles on the WsKG coat are ZnONP dispersed in chitosan. As a result, it was found that the chitosan-ZnONP was well-formed in the coated WsKG. The chitosan-ZnONP coating on fresh-cut papaya has been reported to show antimicrobial activity (Lavinia et al., 2020).

### Shelf life of chitosan-ZnONP coated WsKG

**Fig. 4:**
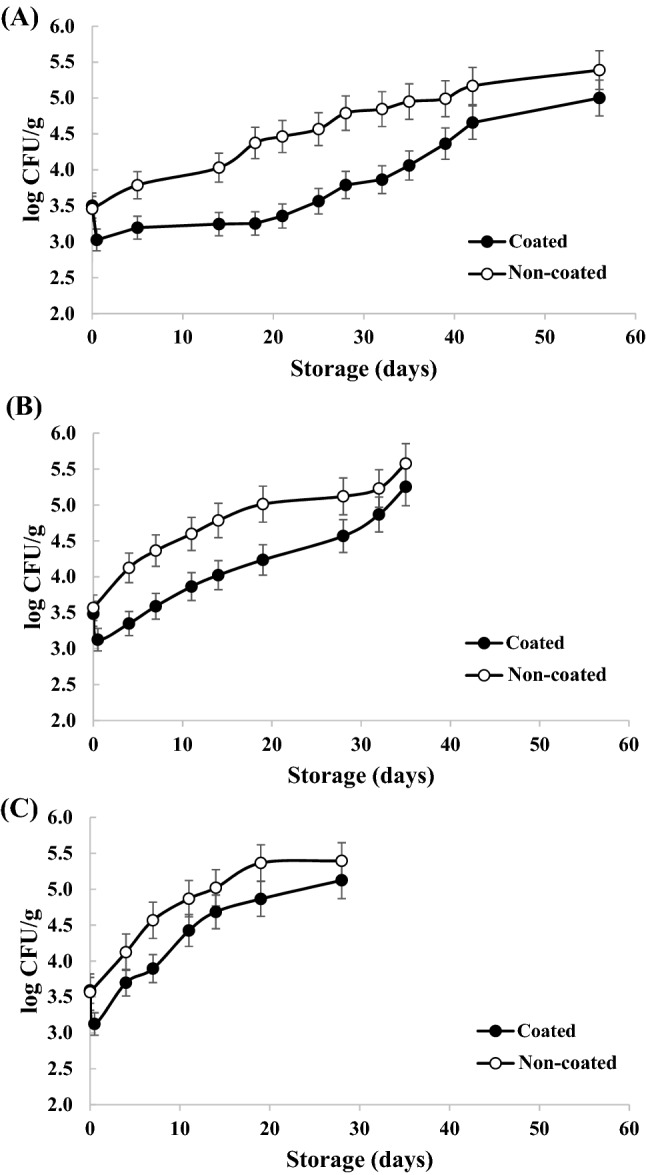
Microbial inhibition (aerobic bacteria) in chitosan-ZnONP coated and non-coated WsKG. (**A**) 5 °C; (**B**) 10 °C; (**C**) 20 °C. Error bars represent the standard deviation (*n* = 3)

The antimicrobial effect on WsKG coated with chitosan-ZnONP (0.03%) solution, which was found to have the greatest antibacterial effect, was analyzed. The growth of microorganisms (total aerobic bacteria) was inhibited in coated WsKG more than non-coated WsKG (Fig. 4). Coated WsKG decreased in the microbial concentration until about 4 h of storage (5, 10, and 20 °C). It shows that the antimicrobial effect was markedly higher than the growth effect in the early stages of growth. Afterwards, the microbial concentration began to increase, which seems to be a phenomenon that appeared as the growth effect increased. At a low temperature of 5 °C, the growth effect was lower than at a high temperature of 20 °C, so it seems that the microbial concentration increased relatively slowly. Overall, the microbial limit of WsKG (4.70 log CFU/g; Ministry of Agriculture, Food and Rural Affairs, 2017) was reached faster as the storage temperature increased. That is, non-coated and coated WsKG showed 3.9 and 6.3 weeks at 5 °C, 1.9 and 4.3 weeks at 10 °C, and 1.3 and 2.0 weeks at 20 °C, respectively. These values were obtained through linear interpolation between data points. Highly reactive oxygen species, such as peroxide ions, hydrogen peroxide, singlet oxygen, and hydroxyl and superoxide radicals, are generated in the presence of ZnONP (Li et al., 2009; Paisoonsin et al., 2013; Tankhiwale and Bajpai, 2012). ZnONP penetrated the cells of the bacteria, thereby damaging protein, DNA, and lipids, destroying the cell membrane or causing cell lysis, resulting in stress-induced production of free radicals (Paisoonsin et al., 2013; Tankhiwale and Bajpai, 2012; Yousef and Danial, 2012). The preservative effect of ZnONP on fruits has been demonstrated such as apple, kiwifruit, mango and papaya (Lavinia et al., 2020; Li et al., 2011; Meng et al., 2014; Meindrawan et al., 2018).

**Fig. 5:**
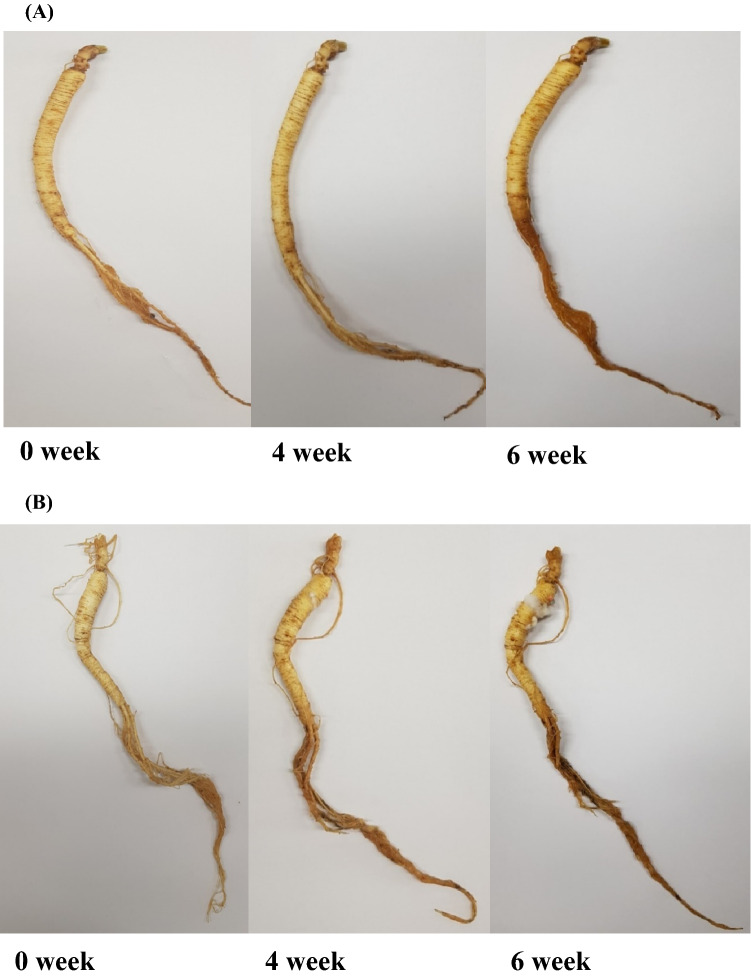
Change in the appearance of (**A**) chitosan-ZnONP coated WsKG and (**B**) non-coated WsKG during storage

The changes in the physical appearance of WsKG at 5 °C, a general refrigeration temperature, are shown in Fig. 5. Mold (white spots at main root) was observed at 4 and 6 weeks for the non-coated WsKG, whereas the coated WsKG did not develop mold for 6 weeks. At 6 weeks, the microbial concentration of aerobic bacteria for the coated WsKG was also below the microbial limit (Fig. 4), indicating that the antimicrobial effect was pronounced. Browning at fine root was developed for the non-coated WsKG more than the coated WsKG. Ginseng turns dark brown by natural fermentation when stored for several days (Bae et al., 2014), and it is more prone to browning at higher temperatures (Hu et al., 2004a, b, 2005). This indicates that the natural fermentation of WsKG was delayed by coating, resulting in the browning inhibition. Overall, the coating could suppress WsKG deterioration.

WsKG is a forest product that is well recognized because of its pharmacological activity. However, WsKG is a perishable crop due to exposure to humid condition from distribution to storage. Therefore, it is important to develop a packaging technique that maintains the freshness of WsKG. In this study, the chitosan-ZnONP coating technique, which has not yet been tried, was applied to WsKG. Result of storage experiments revealed that the quality of coated WsKG was better than the non-coated control in both general bacteria and physical appearance analyses, and the shelf life could be extended to 6 weeks at 5 °C. There are several techniques for extending the shelf life of WsKG, such as modified atmosphere packaging, sanitizer washing, and edible coating with chitosan and organic acids. Through this study, it was found that the chitosan-ZnONP coating technique can also be applied to WsKG. In addition, this chitosan-ZnONP coating technique is expected to work effectively on other perishable produces. However, this study only suggested the applicability of this coating technique to WsKG. In the future, it will be necessary to investigate the effects of the coating variables (coating amount, coating method, etc.) in more details.
